# Deep Brain Stimulation Treating Dystonia: A Systematic Review of Targets, Body Distributions and Etiology Classifications

**DOI:** 10.3389/fnhum.2021.757579

**Published:** 2021-11-26

**Authors:** Houyou Fan, Zijian Zheng, Zixiao Yin, Jianguo Zhang, Guohui Lu

**Affiliations:** ^1^Department of Neurosurgery, The First Affiliated Hospital of Nanchang University, Nanchang, China; ^2^Department of Neurosurgery, Beijing Tiantan Hospital, Capital Medical University, Beijing, China

**Keywords:** deep brain stimulation, dystonia, systematic review, STN (subthalamic nucleus), GPi (globus pallidus internus), body distribution, etiology

## Abstract

**Background:** Deep brain stimulation (DBS) is a typical intervention treating drug-refractory dystonia. Currently, the selection of the better target, the GPi or STN, is debatable. The outcomes of DBS treating dystonia classified by body distribution and etiology is also a popular question.

**Objective:** To comprehensively compare the efficacy, quality of life, mood, and adverse effects (AEs) of GPi-DBS vs. STN-DBS in dystonia as well as in specific types of dystonia classified by body distribution and etiology.

**Methods:** PubMed, Embase, the Cochrane Library, and Google Scholar were searched to identify studies of GPi-DBS and STN-DBS in populations with dystonia. The efficacy, quality of life, mood, and adverse effects were quantitatively compared. Meta-regression analyses were also performed. This analysis has been registered in PROSPERO under the number CRD42020146145.

**Results:** Thirty five studies were included in the main analysis, in which 319 patients underwent GPI-DBS and 113 patients underwent STN-DBS. The average follow-up duration was 12.48 months (range, 3–49 months). The GPI and STN groups were equivalent in terms of efficacy, quality of life, mood, and occurrence of AEs. The focal group demonstrated significantly better disability symptom improvement (*P* = 0.012) than the segmental and generalized groups but showed less SF-36 enhancement than the segmental group (*P* < 0.001). The primary groups exhibited significantly better movement and disability symptom improvements than the secondary non-hereditary group (*P* < 0.005), which demonstrated only disability symptom improvement compared with the secondary hereditary group (*P* < 0.005). The primary hereditary and idiopathic groups had a significantly lower frequency of AEs than the secondary non-hereditary group (*P* < 0.005). The correlation between disability symptom improvement and movement symptom improvement was also significant (*P* < 0.05).

**Conclusion:** GPi-DBS and STN-DBS were both safe and resulted in excellent improvement in efficacy and quality of life in patients with dystonia. Compared with patients with segmental dystonia, patients with focal dystonia demonstrated better improvement in dystonia symptoms but less enhancement of quality of life. Those with primary dystonia had a better response to DBS in terms of efficacy than those with secondary dystonia. Patients who exhibit a significant improvement in movement symptoms might also exhibit excellent improvement in disability symptoms.

## Highlights

This study is by far the most comprehensive and largest-sample meta-analysis of DBS for dystonia.

- GPi-DBS and STN-DBS were equivalent in terms of efficacy, quality of life, mood, and adverse effects.
- Focal dystonia was associated with more disability symptom improvement than segmental and generalized dystonia.
- Primary dystonia was associated with more movement symptom improvement than secondary non-hereditary dystonia.
- Primary dystonia was related to greater disability symptom improvement than secondary dystonia.
- The tolerance of primary hereditary dystonia was better than that of secondary dystonia, while the tolerance of idiopathic dystonia was better than that of secondary non-hereditary dystonia.
- A lower preoperative disability score might be the main predictive factor of higher disability symptom improvement.

## Introduction

Dystonia is a disease that causes the undesired, uncontrollable, and sometimes painful, abnormal movement of an affected limb or body region (Tarsy and Simon, 2006). It is the third most common movement disorder, after Parkinson’s disease and essential tremor, with an estimated overall prevalence of 164 per million individuals (Steeves et al., 2012). This neurological disorder may be classified based on several factors: age at onset, body distribution, temporal pattern, associated features, and etiology (Jinnah and Albanese, 2014). The most widely accepted means by which dystonia is classified are classifications according to body distribution and etiology. Body distribution includes focal dystonia, segmental dystonia, multifocal dystonia, hemidystonia, and generalized dystonia, while etiology accounts for heritability, nervous system pathology and potential idiopathic nature (Albanese et al., 2013). In addition, heritability can be classified further into primary and secondary hereditary (Holloway et al., 2006). This disease has large negative impacts on both the physical and psychological aspects of those affected, including speech, swallowing, writing, feeding, hygiene, dressing, walking, pain, depression, and anxiety (Kupsch et al., 2006; Tsuboi et al., 2019).

In the early 1950s, clinicians treated dystonia with functional surgery in various target sites, including the dentate nucleus, globus pallidus internus (GPi), medial thalamus, and subthalamic nucleus (STN) (Krack and Vercueil, 2001). The first article on deep brain stimulation (DBS) for dystonia was published as early as 1977 (Mundiger, 1977). DBS is a method of intracranial stimulation that uses a controlled direct current that is applied to a specific subcortical nucleus (Montgomery and Gale, 2008). Since that time, many patients have been successfully treated with DBS. However, many differences in methodology, stimulation settings, evaluation, and follow-up have been reported (Benabid et al., 1987; Greene, 2005). Different target nuclei of DBS have been studied in patients with dystonia, including the GPi, the ventrointermediate nucleus (VIM), and the STN (Limousin-Dowsey et al., 1999). The GPi has been typically selected as the primary target for patients with dystonia (Holloway et al., 2006; Gruber et al., 2009), but in recent years, STN-DBS has been suggested to be significantly effective in some types of dystonia and could serve as an alternative for dystonia treatment (Gruber et al., 2009; Ostrem et al., 2011). The selection of an adequate target, the GPi or STN, is still a popular clinical topic that is heavily debated (Brodacki et al., 2017; Zhan et al., 2017; Liu et al., 2019).

Thus far, the efficacy and safety of DBS have been extensively shown in primary generalized (Holloway et al., 2006; Schjerling et al., 2013), segmental (Vidailhet et al., 2005; Holloway et al., 2006), cervical (Gruber et al., 2009; Volkmann et al., 2014), DYT1-positive (Andrews et al., 2010; Borggraefe et al., 2010), myoclonus and tardive dystonia (Welter et al., 2010) in large, well designed, multicenter trials. However, whether this conclusion applies to all types of dystonia is unclear, and whether different types of dystonia respond similarly to DBS is also still controversial. Some people have proposed whether the efficacy of DBS for generalized dystonia is better than for Meige Syndrome because of a larger lesion area, which is more likely to be affected by DBS (Illowsky Karp et al., 1999). It has also been suggested that the treatment of secondary dystonia may be worse than that of primary dystonia because of increased damage in the area of the intracranial lesion (Lumsden et al., 2013). Therefore, a comparison of the efficacy and safety outcomes of DBS for the treatment of dystonia of different classifications is needed. By comparing the results of all types of dystonia, we can draw an overall conclusion on the role of DBS in dystonia.

Here, we performed a meta-analysis to determine the efficacy, quality of life, mood, adverse effects, and possible outcome predictors based on the published literature of STN or GPi DBS for different types of dystonia.

## Methods

### Search Strategy

Our systematic review was conducted following the PRISMA guidelines (Moher et al., 2009). We searched the following databases: PubMed, Embase, Cochrane Central Register of Controlled Trials, Cochrane Movement Disorders Group Trials Register. We also searched citing and cited articles in Google Scholar. The search was limited to human researches published in English. The following keywords were used to perform the search: “dystonia,” “torticollis,” “blepharospasm,” “Meige syndrome,” “deep brain stimulation,” “bilateral,” “globus Pallidus internus,” and “subthalamic nucleus.” The titles, abstracts, full texts, and references were independently screened and assessed by two investigators (FHY and ZZJ). We negotiated together to settle disagreements and reach a consensus.

### Eligibility Criteria

The inclusion criteria for eligible studies were as follows: (1) the study used BFMDRS and/or TWSTRS scores, (2) the study reported the means and standard deviations (SD) of movement or disability BFMDRS/TWSTRS scores, SF-36 scores, and/or BDI scores, (3) the study used bilateral DBS, (4) the study was a randomized, controlled observational or experimental trial, (5) number of patients > 4, and (6) the follow-up duration was longer than 3 months and shorter than 4 years.

The exclusion criteria for eligible studies were: (1) indications for surgery other than dystonia, (2) target other than GPi or STN, (3) staged bilateral or unilateral DBS, (4) DBS with peripheral denervation surgery, (5) studies without outcomes of BFMDRS and/or TWSTRS scores (6) articles that included data that could not be extracted, (7) conference articles, (8) editorials, (9) reviews, (10) case reports, (11) duplicate publications (12) non-English articles.

### Data Extraction and Data Items

A standardized form was used to extract the data. The following information was collected: (1) baseline characteristics of the patients (gender, age at surgery, age at onset, disease duration); (2) operation items (stimulation targets, programming parameters); (3) clinical outcomes (movement and disability BFMDRS/TWSTRS scores, follow-up duration, adverse effects, and other scoring scales at baseline/the last follow-up); and (4) information on body distribution/etiology (determined cases and undefined cases). Discrepancies were resolved by consultations between the authors (FHY, ZZJ).

### Quality Assessment

The Meta-analysis Of Observational Studies in Epidemiology (MOOSE) was used to assess the quality of the studies included in this analysis (Stroup et al., 2000; Phan et al., 2015). Each of the following items was equal to one point, with a maximum of six points: (1) clear study population definition and enough patients (*n* > 10); (2) clear definition and assessment of outcomes; (3) independent evaluation of outcome parameters; (4) clear description of follow-up; (5) no selective loss during follow-up (<10%); and (6) identification of prognostic factors and important confounders. Only studies with a score >5, which were considered methodologically sound, were included in the main analysis. The sensitivity analysis included methodologically unsatisfactory studies. Using this strategy, the main analysis was not affected by unclear and small-sample studies since the sensitivity analysis included all data.

### Meta-Regression

Regression analyses were performed to determine the potential predictors of the efficacy, quality of life, mood, and adverse effects, including age at onset, age at surgery, sex ratio, disease duration, dystonia type, target, and preoperative movement and disability scores.

### Sensitivity Analysis

All studies were included in the sensitivity analysis, although the methodologies of some were less clear (score ≤ 5 in the MOOSE assessment).

### Statistical Analysis

Each study’s effect size was determined by calculating the standardized mean absolute differences (SMD) in movement and disability BFMDRS/TWSTRS scores and 95% confidence intervals (CIs). Adverse events (AEs), including surgery-related, hardware-related, and stimulation-related AEs were recorded to evaluate the safety of DBS for dystonia. The Standard Cochrane Q and I^2^ statistics were used to assess heterogeneity. If *p* < 0.10 or I^2^ > 50%, the data were pooled by a random effect analysis model using a generic-inverse variance. Otherwise, a fixed-effect model was used. The means ± standard error was used as the form of pooled data. Comparisons of the patients’ baseline characteristics between the GPi and STN groups were detected by Student’s *t*-tests. Comparisons of the main outcomes of the two groups, including the surgical effects, quality of life, and adverse effect rates, were also performed using Student’s *t*-tests. *P* < 0.05 indicated a statistically significant difference.

Subgroup analyses of the body distribution and etiology classifications were also performed with total patients’ data (Steeves et al., 2012; Jinnah and Albanese, 2014; Volkmann et al., 2014). Dystonia was categorized as focal, segmental, multifocal, generalized, or hemidystonia based on classification by body distribution. For etiology, the primary hereditary, idiopathic, primary unspecified, secondary hereditary, and secondary non-hereditary groups were used to classify dystonia. Since some patients with primary dystonia lack information about family history or untested primary familial dystonia, another category of “primary unspecified” dystonia was added. Patients with dystonia gene (including DYT1, DYT6, and so on) positive patients were classified as the primary hereditary category. Patients with a negative family history of dystonia and no definite cause of dystonia, as well as primary familial dystonia with dystonia gene negative, were designated as “idiopathic.” Patients with pantothenate kinase-associated neurodegeneration (PKAN), Huntington’s disease (HD), familial myoclonic dystonia (FMD), and Wilson’s disease were classified as the secondary hereditary category. Patients classified as secondary non-hereditary dystonia include patients with cerebral palsy, patients with birth injuries, non-neonatal hypoxia, poststroke/trauma, patients with tardive dystonia, and patients who had other various causes. To detect any differences in the main outcomes, straight pairwise comparisons of the five groups were conducted. The *p*-values were calculated using Student’s *t*-tests and the Bonferroni multiple comparisons correction (Hsu, 1996); *P* < 0.05/N was considered statistically significant, where N was the final number of pairwise comparisons. To estimate the study variance, a simple linear meta-regression based on the unrestricted maximum likelihood model was performed, and *P* < 0.05/N was considered a statistically significant correlation. Comprehensive Meta-Analysis 2.2 (Biostat, Englewood, NJ, United States) and Stata 12.0 (Stata Corp, College Station, TX, United States) were both used to perform the statistical analyses. The data were managed using the Meta-analysis of Observational Studies in Epidemiology (MOOSE) Group and the Cochrane Handbook for Systematic Reviews of Interventions (Stroup et al., 2000; Higgins and Green, 2011). This analysis has been registered in PROSPERO under the number CRD42020146145.

## Results

### Search Results

**FIGURE 1:**
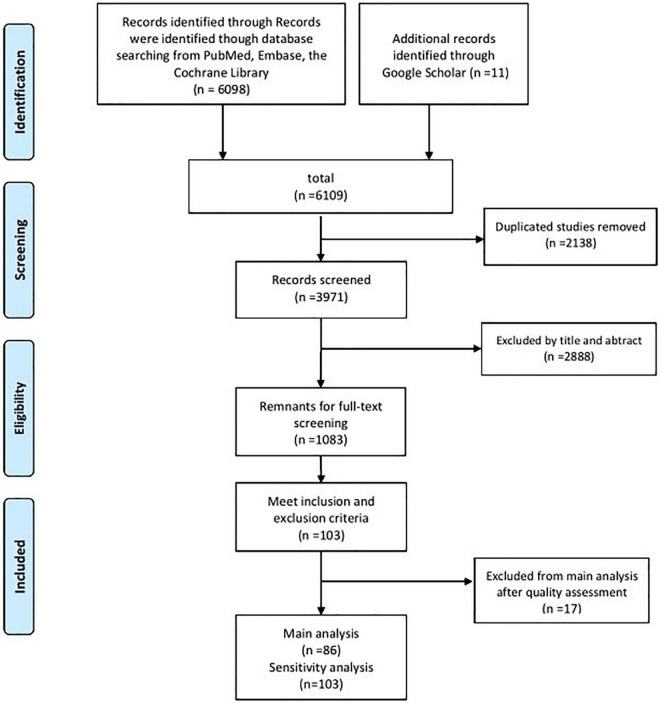
PRISMA flow chart of the studies included in the main analysis and the sensitivity analysis.

According to the keyword search, 6,109 articles were identified. After duplicate articles were removed and titles and abstracts were filtered, 3,971 articles were excluded. The reasons for exclusion were that they were studies unrelated to dystonia, non-clinical studies, and low-quality articles (conference articles, letters, editorials, professional opinions, and case reports). According to the inclusion and exclusion criteria, the remaining 1,083 articles were secondarily screened by reading the full texts. The references of these studies were also screened. Finally, 103 studies that met all the criteria were filtered out for the MOOSE quality assessment. The specific screening process is illustrated in Figure 1.

### Quality Assessment

Based on the MOOSE quality assessment, 70% of the studies lost points due to insufficient patient numbers or insufficient outcome parameters (Supplementary Table 1). Overall, the methodology of 68 articles, which included 140 patients, was considered not clear enough. Therefore, these articles were eliminated from the main analysis. The specific data and other information of all 103 articles are shown in Supplementary Table 1.

### Baseline Characteristics and Treatment Efficacy

The baseline characteristics, including age at onset, age at surgery, and length of follow-up, were not significantly different between the groups. However, the disease duration in the GPi group was higher than that in the STN group (Table 1).

**TABLE 1:** Pooled value of patient baseline characteristics^a^.

	GPi-DBS	STN-DBS	*P*-value
Age of surgery	37.85 ± 2.05 (319)	42.26 ± 6.61 (125)	0.55
Age at onset	25.01 ± 1.19 (208)	38.74 ± 7.57 (58)	0.14
Disease duration (years)	10.69 ± 0.85 (192)	4.64 ± 1.03 (102)	**0.01**
Follow-up duration (months)	10.08 ± 3.53 (207)	9.84 ± 2.06 (102)	0.50

*GPi, globus pallidus internus; STN, subthalamic nucleus.*

*^a^“Mean ± standard error (number of observations)” is used to represent the data, and the comparisons with significant differences are highlighted.*

### Main Outcomes of Deep Brain Stimulation Efficacy

#### Globus Pallidus Internus vs. Subthalamic Nucleus

The two groups were equivalent in movement symptom improvement, disability symptom improvement, SF-36 increase, BDI enhancement and adverse effect rates (AERs) (Table 2). However, in the efficacy assessment of each intervention, forest plots showed significant postoperative movement improvement for both GPi-DBS (standardized mean difference = 1.56; 95% CI:1.39–1.72; *P* < 0.001) and STN-DBS (standardized mean difference = 2.06; 95% CI:1.32–2.81; *P* < 0.001). Disability scores also improved significantly for both GPi-DBS (standardized mean difference = 1.09; 95% CI = 0.91–1.28; *P* < 0.001) and STN-DBS (standardized mean difference = 1.64; 95% CI = 1.89–2.39; *P* < 0.001).

**TABLE 2:** The main and sensitivity analyses of the efficacy, quality of life and adverse effects after DBS*^a^*.

	GPi-DBS	STN-DBS	*P*-value
**Main analysis**			
Movement scores (SMD)	1.56 ± 0.08 (319)	2.06 ± 0.35 (106)	0.12
Disability scores (SMD)	1.09 ± 0.09 (286)	1.64 ± 0.09 (113)	0.10
PI SF-36	43.33 ± 18.33 (29)	31.62 ± 7.09 (16)	0.77
PI BDI	25.92 ± 6.12 (39)	16.26 ± 45.76 (7)	0.58
Adverse effects (%)	23.3 ± 3.4 (133)	34.0 ± 13.3 (41)	0.53
**Sensitivity analysis**			
Movement scores (SMD)	1.65 ± 0.08 (1349)	2.11 ± 0.35 (125)	0.20
Disability scores (SMD)	1.2 ± 0.09 (998)	1.25 ± 0.34 (123)	0.24
PI SF-36	25.69 ± 4.8 (39)	32.83 ± 7.09 (20)	0.44
PI BDI	30.54 ± 6.33 (172)	29.24 ± 12.7 (16)	0.47
Adverse effects (%)	24.1 ± 3.4 (519)	34.0 ± 13.4 (41)	0.57

*DBS, deep brain stimulation; SMD, standardized mean difference; PI, percentage improvement postoperatively; SF-36, 36-item Short Form Health Survey; BDI, Beck Depression Inventory.*

*^a^“Mean ± standard error (number of observations)” is used to represent the data. The outcomes are demonstrated by p-values and the comparisons with significant differences are highlighted.*

The improvements in SF-36 and BDI scores were 43.33 and 25.92%, respectively, for GPi-DBS and 31.62 and 16.26%, respectively, for STN-DBS. The pooled AERs after GPi-DBS and STN-DBS were 23.3 and 34.0%, respectively (Table 2).

#### Subgroup Analysis of Body Distributions

Of the studies included in this subgroup analysis, 13 were in the focal group, 41 were in the generalized group, 16 were in the segmental group, 2 were in the multifocal group, and none was in the hemidystonia group. Due to the lack of available data, the outcomes of the multifocal and hemidystonia groups could not be analyzed. The movement scores, disability scores, and the SF-36 and BDI scores after DBS all showed significant improvement in the remaining three groups (Table 3). No significant difference was observed among the three groups in terms of movement symptom improvement, BDI enhancement, or AERs (Table 3).

**TABLE 3:** The main and sensitivity analyses of the efficacy, quality of life and adverse effects after DBS in the body distribution subgroup*^a^*.

	Focal	Segmental	Generalized	Focal vs. Seg	Focal vs. Gen	Seg vs. Gen
**Main analysis**						
Movement scores (SMD)	2.1 ± 0.24 (146)	1.44 ± 0.20 (132)	1.47 ± 0.12 (553)	0.09	0.02	0.82
Disability scores (SMD)	1.7 ± 0.25 (122)	0.8 ± 0.24 (91)	1.0 ± 0.14 (420)	**0.01**	**0.01**	0.53
PI SF-36	18.12 ± 6.68 (16)	37.3 ± 3.76 (10)	/	**0.01**	/	/
PI BDI	28.77 ± 6.14 (45)	/	25.14 ± 6.33 (73)	/	/	0.70
Adverse Effects (%)	43.6 ± 12.4 (75)	17.7 ± 7.9 (60)	24.5 ± 9.0 (53)	0.29	0.22	0.57
**Sensitivity analysis**						
Movement scores (SMD)	2.41 ± 0.2 (231)	1.44 ± 0.23 (171)	1.49 ± 0.11 (559)	**0.01**	**0.01**	0.83
Disability scores (SMD)	2.0 ± 0.21 (174)	0.9 ± 0.23 (101)	1.0 ± 0.14 (426)	0.01	**0.01**	0.746
PI SF-36	16 ± 1.67 (26)	37.3 ± 3.76 (10)	/	**0.01**	/	/se
PI BDI	35.4 ± 10.65 (64)	16.26 ± 6.33 (7)	25.14 ± 6.33 (73)	0.336	0.528	0.700
Adverse effects (%)	42.4 ± 8.6 (110)	17.3 ± 9.6 (60)	22.8 ± 8.1 (53)	0.265	0.186	0.571

*DBS, deep brain stimulation; SMD, standardized mean difference; PI, percentage improvement postoperatively; SF-36, 36-item Short Form Health Survey; BDI, Beck Depression Inventory.*

*^a^“Mean ± standard error (number of observations)” is used to represent the data. The outcomes are demonstrated by p-values and the comparisons with significant differences are highlighted.*

The segmental and generalized groups demonstrated significantly less disability symptom improvement than the focal group, while no differences were observed between the segmental and generalized groups. In addition, the segmental group exhibited a significantly better SF-36 enhancement than the focal group. Notably, the SF-36 data in the generalized group and the BDI data in the segmental group were not available.

#### Subgroup Analysis of Etiology

In this subgroup analysis, 68 studies containing 908 patients were included (Table 4). Due to the lack of available data, the percentage of postoperative improvement in SF-36 and BDI scores could not be analyzed. The movement and disability scores both showed significant improvement in the five groups (Table 4). The secondary non-hereditary group demonstrated significantly less movement symptom improvement than the primary hereditary, idiopathic, and primary unspecified groups (*p* < 0.005), while no differences were observed between the other groups by pairwise comparisons. For disability symptoms, the secondary hereditary and secondary non-hereditary groups both showed significantly less improvement than the primary hereditary, idiopathic, and primary unspecified groups (*p* < 0.005). The frequency of AEs in the primary hereditary and idiopathic groups was significantly lower than that in the primary unspecified and secondary non-hereditary groups, while significant differences were also observed between the primary hereditary and secondary hereditary groups (*p* < 0.005).

**TABLE 4:** The main and sensitivity analyses of the surgery-related outcomes after DBS in the etiology subgroup*^a^*.

	Primary hereditary (PH)	Idiopathic (ID)	Primary unspecified (PU)	Secondary hereditary (SH)	Secondary non-hereditary (SN)	PC outcomes^b^
**Main analysis**						
Movement scores (SMD)	2.03 ± 0.17 (319)	1.69 ± 0.16 (202)	1.96 ± 0.20 (178)	0.95 ± 0.44 (29)	0.77 ± 0.13 (152)	+++^c^
Disability scores (SMD)	1.54 ± 0.16 (206)	1.21 ± 0.19 (216)	1.42 ± 0.245 (128)	0.43 ± 0.19 (57)	0.57 ± 0.15 (129)	++++++^d^
Adverse effects (%)	6.6 ± 3.3 (59)	9.0 ± 6.4 (22)	38.0 ± 5.5 (124)	21.2 ± 7.4 (38)	60.9 ± 16.3 (23)	+++++^e^
**Sensitivity analysis**						
Movement scores (SMD)	1.96 ± 0.14 (326)	1.76 ± 0.16 (338)	2.17 ± 0.29 (216)	0.95 ± 0.44 (29)	0.94 ± 0.16 (171)	+++^f^
Disability scores (SMD)	1.54 ± 0.16 (207)	1.33 ± 0.19 (232)	1.58 ± 0.225 (158)	0.43 ± 0.19 (57)	0.65 ± 0.14 (148)	++++++^g^
Adverse effects (%)	6.5 ± 4.1 (59)	15.7 ± 6.1 (47)	37.9 ± 5.6 (134)	21.5 ± 7.7 (38)	60.9 ± 16.6 (23)	+++++^h^

*DBS, deep brain stimulation; PC, pairwise comparison; SMD, standardized mean difference.*

*^a^“Mean ± standard error (number of observations)” is used to represent the data.*

*^b^Each significant comparison is marked as a “+.”.*

*^c^p-value of the pairwise comparison of “Movement scores (SMD)” in the main analysis: PH vs. SN < 0.005; ID vs. SN < 0.005; PU vs. SN < 0.005.*

*^d^p-value of the pairwise comparison of “Disability scores (SMD)” in the main analysis: PH vs. SH < 0.005; ID vs. SH < 0.005; PU vs. SH < 0.005; PH vs. SN < 0.005; ID vs. SN < 0.005; PU vs. SN < 0.005.*

*^e^p-value of the pairwise comparison of “Adverse Effects (%)” in the main analysis: PH vs. PU < 0.005; PH vs. SH < 0.005; PH vs. SN < 0.005; ID vs. PU < 0.005; ID vs. SN < 0.005.*

*^f^p-value of the pairwise comparison of “Movement scores (SMD)” in the sensitivity analysis: PH vs. SN < 0.005; ID vs. SN < 0.005; PU vs. SN < 0.005.*

*^g^p-value of the pairwise comparison of “Disability scores (SMD)” in the sensitivity analysis: PH vs. SH < 0.005; ID vs. SH < 0.005; PU vs. SH < 0.005; PH vs. SN < 0.005; ID vs. SN < 0.005; PU vs. SN < 0.005.*

*^h^p-value of the pairwise comparison of “Adverse Effects (%)” in the sensitivity analysis: PH vs. PU < 0.005; PH vs. SH < 0.005; PH vs. SN < 0.005; ID vs. PU < 0.005; ID vs. SN < 0.005.*

### Meta-Regression

**FIGURE 2:**
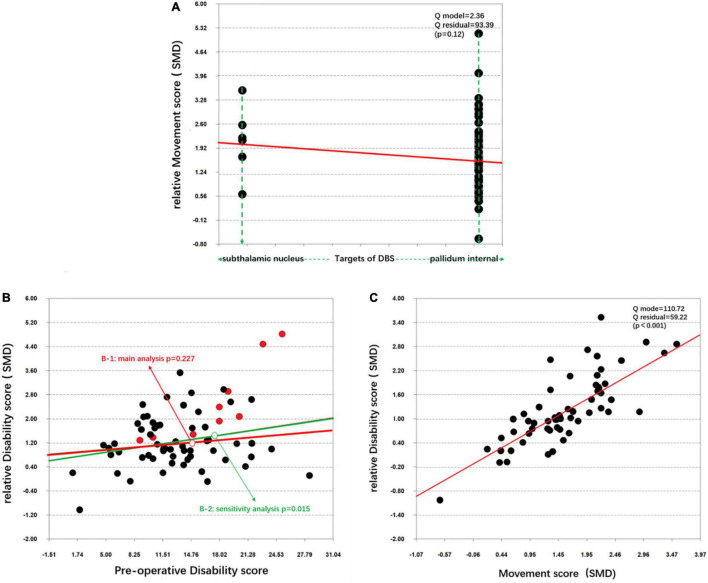
Simple linear regression of the target differences and disability symptom improvement. **(A)** Correlation between the target differences and the relative movement score (SMD). **(B)** Correlation between the preoperative disability score and the relative disability score (SMD) in the main analysis (B-1) and the sensitivity analysis (B-2). **(C)** Correlation between the movement score (SMD) and relative disability score (SMD).

Through a simple linear regression analysis, we found that age at onset (*p* = 0.191), disease duration (*p* = 0.553), age at surgery (*p* = 0.154) and preoperative movement scores (*p* = 0.105) were not significant predictors of movement symptom improvement. They were also not significant predictors of disability symptom improvement, SF-36 score improvement, BDI enhancement, or AERs. A significant correlation was observed between disability symptom improvement and movement symptom improvement (Figure 2).

### Sensitivity Analysis

The 17 excluded articles were added, and all 103 cohorts were used to re-pool the data. Although the statistical values were slightly changed (Tables 2–4), the statistical significance of the data did not change for most outcomes with three exceptions. The focal group demonstrated significantly better movement symptom improvement than the segmental and generalized groups. The generalized group exhibited significantly less disability symptom improvement than the focal group (Table 3). The correlation between the preoperative movement scores and disability symptom improvement was also significant (Figure 2B-2).

## Discussion

In recent years, several comprehensive literature reviews on DBS for dystonia have been published (Holloway et al., 2006; Koy et al., 2013; Vidailhet et al., 2013; Elkaim et al., 2019; Girach et al., 2019; Rodrigues et al., 2019). However, none of these reviews have compared the differences in efficacy and quality of life outcomes between GPi-DBS and STN-DBS, which are the two most common clinical targets for dystonia. Due to the various classification systems for dystonia, the two most widely accepted classification schemes were chosen to pool the data to allow subgroup analyses of body distribution and etiology. This meta-analysis represents a method of obtaining a reasonable understanding of the effect of DBS on a complex syndrome (dystonia). The efficacy of DBS directed at two targets, the GPi and STN, was not significantly different in our meta-analysis. Moreover, our study indicated that the focal group exhibited significantly better disability symptom improvement but less SF-36 enhancement than the segmental group. All primary groups performed significantly better in terms of movement and disability symptom improvements than the secondary non-hereditary group, which demonstrated better disability symptom improvement compared with the secondary hereditary group. The primary hereditary and idiopathic groups had a significantly lower frequency of AEs than the secondary non-hereditary group. The correlation between disability symptom improvement and movement symptom improvement was also significant.

### The Target of Deep Brain Stimulation

Overall, both GPi-DBS and STN-DBS patients showed statistically significant improvements in movement symptoms, disability symptoms, SF-36 scores, and BDI scores in our analysis. Though the mean movement scores (SMD) and disability scores (SMD) in the STN group were numerically higher than those in the GPi group, they were not significantly different. To our knowledge, the clinical outcomes of GPi-DBS and STN-DBS in patients with dystonia have been evaluated in two other studies (Schjerling et al., 2013; Lin et al., 2019). The authors proposed that both GPi and STN targets are effective in treating dystonia; however, the extent of the movement and disability improvements was substantially larger in the STN group, which is comparable to the findings of this study. Schjerling et al. (2013) reported that: after stimulation of the STN, the mean 6-month improvement in BFMDRS movement score was 13.8 points; after stimulation of the GPi, this improvement was 9.1 points. Lin et al. (2019) also reported that the percentage improvement in the BFMDRS total movement score was significantly larger after STN DBS (64%) than after GPi DBS (48%) after the 12-month follow-up.

In contrast, the mean percentage of postoperative improvements (PIs) in the SF-36 and BDI scores in the GPi group was numerically higher than that of the STN-DBS group, but the differences were not statistically significant. According to multiple researchers, GPi-DBS improves the quality of life in patients with dystonia (Schjerling et al., 2013; Girach et al., 2019; Liu et al., 2019). However, few studies have investigated the quality of life in patients with dystonia who undergo STN-DBS (Lin et al., 2019). According to a follow-up assessment by Lin and Elkaim et al., no significant difference was observed between the groups in terms of the percentage improvement in quality of life (Elkaim et al., 2019; Lin et al., 2019).

Depression could be regarded as one type of stimulation-related AEs (Liu et al., 2019). Both STN-DBS and GPi-DBS could cause transient depression. In addition, both can result in surgery-related adverse effects, including hemorrhages and infections. Although GPi-DBS is known to have a direct influence on dyskinesia, STN-DBS is thought to be effective in these patients because of its effect on Parkinson’s symptoms, which leads to a substantial reduction in medication use, thus avoiding hyperkinesia (Dinkelbach et al., 2015). In our present study, the pooled AER after GPi-DBS was numerically lower than that after STN-DBS, but the difference was not significant. This outcome might partly be due to the different stimulation parameters used in GPi-DBS and STN-DBS considering the stimulation-related AEs (Tsuboi et al., 2020). More studies are needed to explore the relationship between programming parameters and safety.

### Subgroup Analysis of Body Distribution

Here, we focused on the focal, segmental, and generalized groups. The movement scores, disability scores, and the SF-36 and BDI scores after DBS all showed significant improvement in the three groups. The movement improvement for focal dystonia was slightly higher than segmental and generalized dystonia with no statistical difference. The segmental and generalized groups demonstrated significantly less disability improvement than the focal group, while no differences were observed between the segmental and generalized groups. A 42.9% improvement in dystonia, as assessed by the dystonia movement score, and a 63.8% improvement, as assessed by the dystonia disability score, were demonstrated by the first prospective, multicenter, single-blind study that assessed the efficacy and safety of DBS in cervical dystonia (Ostrem et al., 2007). In generalized and segmental dystonia, two double-blind and multicenter studies demonstrated a benefit ratio, with mean improvements in the dystonia movement score of 51 and 42% (Steeves et al., 2012; Zhan et al., 2017). Although patients with focal, segmental and generalized dystonia all exhibited good responses to DBS, patients with focal dystonia might demonstrate the most obvious improvement in motor symptoms and disability symptoms. The physiological and pathological mechanisms of dystonia and DBS might contribute to this phenomenon (Krauss, 2002; Kiss et al., 2007; Levinson et al., 2021).

### Subgroup Analysis of Etiology

Based on etiology, dystonia is classified as either primary hereditary, idiopathic, primary unspecified, secondary hereditary, or secondary non-hereditary. Primary unspecified means the genetic tests of those patients were unclear. The movement and disability scores both showed significant improvement in the five groups. We observed that all the primary groups demonstrated significantly better movement and disability symptom improvements than the secondary non-hereditary group, which showed greater disability symptom improvement than the secondary hereditary group. However, the mean values of movement symptom improvements in the primary groups were all higher than those of the secondary hereditary group. In previous studies, many authors noted a significant benefit of DBS in patients with primary dystonia (Benabid et al., 1987; Koy et al., 2013; Filip et al., 2017). Specifically, the dramatic response to DBS was shown in dystonia musculorum deformans-1 (DYT1 +) patients, by Markun et al. and other researchers (Reese et al., 2011; Cao et al., 2013; Quartarone and Hallett, 2013). Previous studies also noted that patients with primary dystonia exhibited a better response to DBS than those with secondary dystonia (Markun et al., 2012), a conclusion that is comparable with the outcomes of this study.

The frequency of AEs in the primary hereditary and idiopathic groups was significantly lower than that in the primary unspecified and secondary non-hereditary groups, and there were also significant differences between the primary hereditary and secondary hereditary groups. However, these results should be interpreted with caution. Although we analyzed 16 clinical studies in this analysis, the idiopathic group only included 22 patients from two studies, while the secondary non-hereditary group only included 23 patients from one study. Therefore, the statistical calculations containing these two groups should be carefully interpreted. Secondary hereditary dystonia includes pantothenate kinase–associated neurodegeneration (PKAN), Wilson’s disease, and Huntington’s disease (HD). Panov et al. (2013) proposed that the AER in PKAN patients was 23.2%, and the AER in the secondary hereditary group was 21.2 ± 7.4% in the current study. Due to the dramatic response of primary hereditary dystonia (Cif et al., 2010; Cao et al., 2013; Quartarone and Hallett, 2013), its AER was also much lower compared with other types of dystonia.

### Prognostic Factors

In this study, we attempted to determine the prognostic factors for DBS as a dystonia treatment. Compared with the baseline characteristics, it was difficult to compare the efficacy and safety of DBS between the GPI-DBS and STN-DBS groups because the disease duration was significantly different between these groups. However, no statistically significant change was observed in the efficacy or safety of DBS across the time of disease duration. Moreover, age at surgery, age at onset, and preoperative scores were also not significant predictors of movement symptom improvement, disability symptom improvement, SF-36 enhancement, BDI enhancement, or AERs. Nevertheless, we found patients who demonstrated significant improvements in movement symptoms likely also demonstrated excellent improvement in disability symptoms.

We cannot directly deny that these factors may contribute to a difference in the outcomes of patients with dystonia, and many studies have attempted to determine the possible prognostic factors for DBS in the treatment of different types of dystonia. In a previous study, Isaias et al. (2011) found that a younger age at surgery (<21 years of age) and shorter disease duration (<15 years) are the main predictive factors of good postoperative outcomes for primary dystonia (Eltahawy et al., 2004; De Vloo et al., 2019). Brüggemann et al. (2015) observed that caudate atrophy was a predictor of a less beneficial outcome. Rodrigues et al. (2019) recently showed that cortical plasticity can be used as a biomarker to verify outcomes of DBS treatment, which indicates a positive effect of DBS. Furthermore, Isaias et al. (2011) found that worse baseline severity is the main predictive factor of higher efficacy of DBS for the treatment of Meige syndrome. Actually, in our sensitivity analysis, the correlation between the preoperative disability score and disability symptom improvement was significant, which might be related to the floor effect. A floor effect, also known as a basement effect, means that when there is a certain improvement level, patients with mild symptoms may not have as much room for improvement as patients with severe symptoms (Isaias et al., 2011). However, we need to be cautious to conclude that a lower preoperative disability score is the main predictive factor of higher disability symptom improvement for all patients with dystonia. More convincing clinical trials are therefore needed.

### Limitations

Our study had several limitations.

First, most of the included studies were not randomized controlled studies, and only two studies contained comparisons of the two types of DBS, which had some advantages and some disadvantages. The disadvantage was high heterogeneity, which introduced biases and reduced the evidence level. The advantage was that a large number of patient samples from multiple centers was included in this study, which increased the statistical validity and universality of the results. Actually, valuable clinical information can also be provided by the one-arm meta-analysis (Weaver et al., 2005; Yin et al., 2019; Giordano et al., 2020).

Second, the number of patients treated with STN-DBS was not sufficient, and additional studies of patients treated with STN-DBS are therefore needed.

Third, SF-36 was used as the Qol tool in our analysis. Though it’s widely used and can evaluate patients’ health as a whole, it contains very few problems with dystonia.

Fourth, the follow-up duration (mean of 12.5 months) was short. Therefore, the long-term impacts and safety data could not be pooled or calculated.

Finally, in terms of the methodology, included studies were limited to those published in English and excluded some older articles that could not be retrieved.

## Conclusion

This meta-analysis demonstrated favorable outcomes in terms of efficacy, quality of life, and safety. GPi-DBS and STN-DBS were both safe to perform and efficacious, and both resulted in excellent improvement in the quality of life of patients with dystonia. Compared with patients with segmental dystonia, those with focal dystonia exhibited a better improvement in dystonia symptoms but exhibited less enhancement in quality of life. Those with primary dystonia had a better response to DBS in terms of efficacy than those with secondary dystonia. Patients who demonstrated a significant improvement in movement symptoms likely also demonstrated excellent improvement in disability symptoms. Additional outcome data for patients treated with STN-DBS are needed. Collectively, we believe that results from future studies would enable clinicians to provide patients with a clearer perspective and to enhance the efficacy, quality of life, and safety as they relate to DBS.

## Data Availability Statement

The original contributions presented in the study are included in the article/Supplementary Material, further inquiries can be directed to the corresponding author/s.

## Author Contributions

GL contributed to the concept and design of this manuscript. JZ modified the study design. HF and ZZ collected data, finished the manuscript, and contributed equally to this work. ZY helped the statistical and plotting process. All authors contributed to the article and approved the submitted version.

## Conflict of Interest

The authors declare that the research was conducted in the absence of any commercial or financial relationships that could be construed as a potential conflict of interest.

## Publisher’s Note

All claims expressed in this article are solely those of the authors and do not necessarily represent those of their affiliated organizations, or those of the publisher, the editors and the reviewers. Any product that may be evaluated in this article, or claim that may be made by its manufacturer, is not guaranteed or endorsed by the publisher.
